# A community-based nurse-led medication self-management intervention in the improvement of medication adherence in older patients with multimorbidity: protocol for a randomised controlled trial

**DOI:** 10.1186/s12877-021-02097-x

**Published:** 2021-03-02

**Authors:** Chen Yang, Zhaozhao Hui, Dejian Zeng, Song Zhu, Xiuhua Wang, Diana Tze Fan Lee, Sek Ying Chair

**Affiliations:** 1 The Nethersole School of Nursing, Faculty of Medicine, The Chinese University of Hong Kong, 6/F, Esther Lee Building, Shatin, Hong Kong SAR China; 2 Department of Thoracic Surgery, The Second Xiangya Hospital of Central South University, Changsha, China; 3 Xiang Ya Nursing School, Central South University, Changsha, China

**Keywords:** Medication adherence, Multimorbidity, Self-management, Older patients

## Abstract

**Background:**

Older patients suffering from multimorbidity are at high risk of medication nonadherence. It has been well established that self-management support is an effective strategy to enhance medication adherence for patients with chronic conditions. However, little is known about the effect of the medication self-management intervention in older patients with multimorbidity. This paper presents the protocol for a study that aims to evaluate the effectiveness of a nurse-led medication self-management intervention in improving medication adherence and health outcomes for community-dwelling older patients with multimorbidity.

**Methods:**

The study protocol follows the recommendations of the Standard Protocol Items: Recommendations for Interventional Trials 2013 statement. This study is a multicentre, single-blind, two-arm randomised controlled trial. Older patients with multimorbidity will be recruited from three community health centres in Changsha, China. A total of 136 participants will be randomly allocated to receive usual care or usual care plus the medication self-management intervention. The intervention will be delivered by community nurses. The 6-week intervention includes three face-to-face education sessions and two weekly follow-up phone calls. Participants in the control group continue to receive all respects of usual care offered by community healthcare providers, including chronic disease management, drug prescription, referral to hospital specialists, health education and consultations regarding patients’ diseases and treatments during centre visits. The primary outcome is medication adherence as measured by the 5-item Medication Adherence Report Scale. Secondary outcomes include medication self-management capacity (medication knowledge, medication beliefs, medication social support, medication skills, and medication self-efficacy), treatment experiences (medication treatment satisfaction and treatment burden), quality of life, and utilisation of healthcare services. All outcomes will be measured at baseline, immediately post-intervention, and at 3-month post-intervention.

**Discussion:**

This study will provide evidence about the effectiveness of a medication self-management intervention, delivered by nurses, for older patients with multimorbidity and adherence problems. It is expected that the results of the study, if proven effective in improving patients’ adherence and health outcomes, will provide evidence-based self-management support strategies for healthcare providers in routine chronic disease management in community settings.

**Trial registration:**

The trial is registered at ChiCTR.org.cn (ChiCTR2000030011; date February 19, 2020).

**Supplementary Information:**

The online version contains supplementary material available at 10.1186/s12877-021-02097-x.

## Background

Adherence is the key factor to reach the potential efficacy of medication treatments for patients with chronic conditions. However, according to the World Health Organization, it is estimated that 50% of these patients are not adherent to medication regimens in developed countries [1]. The number is assumed to be higher in developing countries, given limited access to healthcare services and paucity of medical providers and finances. Medication nonadherence has been frequently reported to be associated with adverse clinical outcomes [2], decreased quality of life [3], as well as higher utilisation of healthcare resources [4].

Medication nonadherence is highly prevalent in older populations, and more than 55% of them are living with multimorbidity (i.e., the coexistence of multiple chronic diseases) [5]. Along with a high level of complexity, older patients with multimorbidity are more likely to experience complex medication regimens [6], adverse drug events [7], and substantial drug burden [8]. Patients have to consume considerable energy and time to take and manage their medications [9, 10]. The presence of multimorbidity has been identified as a barrier to self-management of medications and diseases, and thus leads to poor medication adherence and treatment outcomes [11, 12].

Medication self-management support is an effective strategy to address challenges in medication-taking and empower patients to self-care. Generally, a medication self-management program integrates multiple components aimed at helping patients to effectively and safely take medications, such as health behaviour change, patient education, shared decision making, and goal setting. The positive effects of medication self-management interventions have been established across multiple conditions [13–15]. However, current clinical trials mainly targeted at a single condition or medication, which could limit the generalizability of their findings to older patients with multimorbidity [16, 17]. Just a few studies, to the best of our knowledge, explored the effectiveness of the self-management intervention for this complex group of patients [18–21]. Unfortunately, most of them failed to yield significant improvements in adherence or health outcomes. A 6-month multimorbidity self-management support program based on cognitive behavioural therapy and motivational interviewing found no improvements in any patient outcomes except self-rated health [18]. Another nurse-led personalised self-management care intervention for patients with coronary heart disease and depression also reported no significant differences in adherence and health outcomes between the intervention and control group [19]. One likely explanation is that most self-management programmes for older patients with multimorbidity were designed to cope with each aspect of self-care, but not specifically to improve medication self-management capacity or focus on patients with adherence problems. A Cochrane review concluded that interventions are more likely to be effective when targeting specific problems experienced by patients with multimorbidity, such as depression, functional limitation, and medication nonadherence [22]. Interventions that have broader focus, such as case management or changes in care delivery for patients with multimorbidity, seems less effective. The authors also suggested that a medication management intervention targeting patients’ specific problems in medication use is more effective. In addition, the social behavioural models were rarely used to guide the study design and to explain the underlying mechanisms of significant or non-significant intervention effects in previous studies. Therefore, although there is growing interest in research for older patients with multimorbidity, promoting medication adherence of this group of patients is still a challenge for healthcare providers and researchers [23]. The current study will implement an evidence-based, theory-informed, and nurse-led medication self-management intervention among older patients with multimorbidity and examine its effects in community settings.

### Study objectives and hypotheses

The primary objective is to evaluate the effectiveness of a six-week nurse-led medication self-management intervention on the improvement of medication adherence in community-dwelling older patients with multimorbidity, as compared with usual care. Secondary objectives are to evaluate the effectiveness of the intervention on the improvement of patients’ medication self-management capacity (medication knowledge, medication beliefs, medication social support, medication skills, and medication self-efficacy), treatment experiences (medication treatment satisfaction and treatment burden), quality of life, and utilisation of healthcare services, as compared with usual care.

The primary hypothesis is that the participants in the intervention group will have better medication adherence at 3 months after the intervention compared to those in the control group. The secondary hypotheses are that the participants in the intervention group, will have (1) better medication adherence, medication self-management capacity, treatment experiences, and quality of life immediately after the intervention compared to participants in the control group, and (2) better medication self-management capacity, treatment experiences, and quality of life, as well as fewer utilisation of healthcare services at 3 months after the intervention compared to those in the control group.

## Methods

### Trial design

**Fig. 1:**
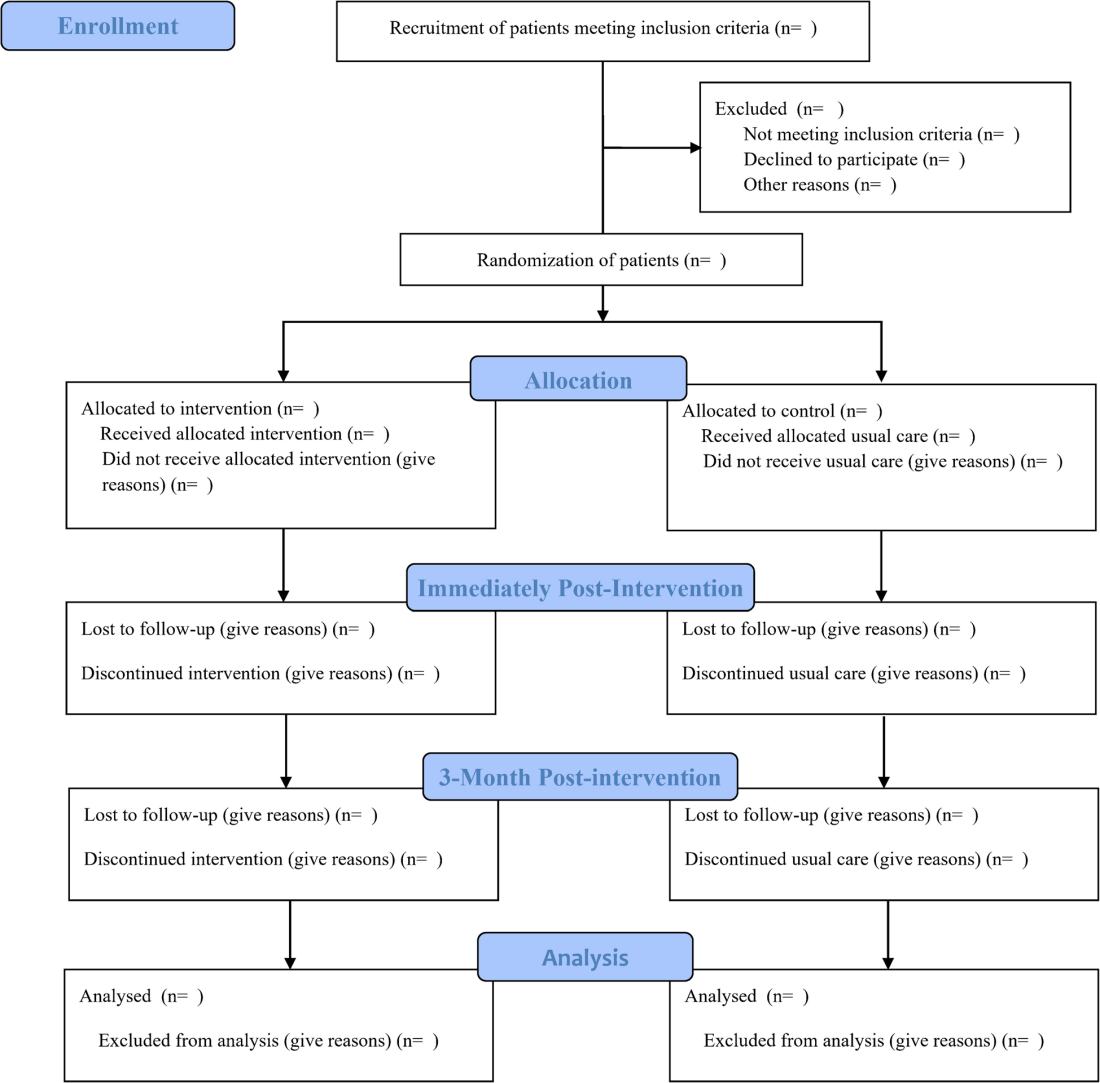
CONSORT flow diagram of the study protocol

This is a multicentre, single-blind, two-arm randomised controlled trial with randomisation at the participant level. Participants will be randomly allocated to receive usual care (control) or usual care plus the medication self-management intervention (intervention). Primary and secondary study outcomes will be collected for the intervention group and control group at baseline (T0), in the week immediately post-intervention (T1), and at 3-month post-intervention (T2). The trial has been registered at ChiCTR.org.cn (ChiCTR2000030011). This protocol is presented based on the recommendations of the Standard Protocol Items: Recommendations for Interventional Trials (SPIRIT) 2013 statement [24]. The results of this trial will be reported as per the Consolidated Standards of Reporting Trials (CONSORT) 2010 statement [25]. An overview of the study design is shown in Fig. 1.

### Theoretical framework

**Fig. 2:**
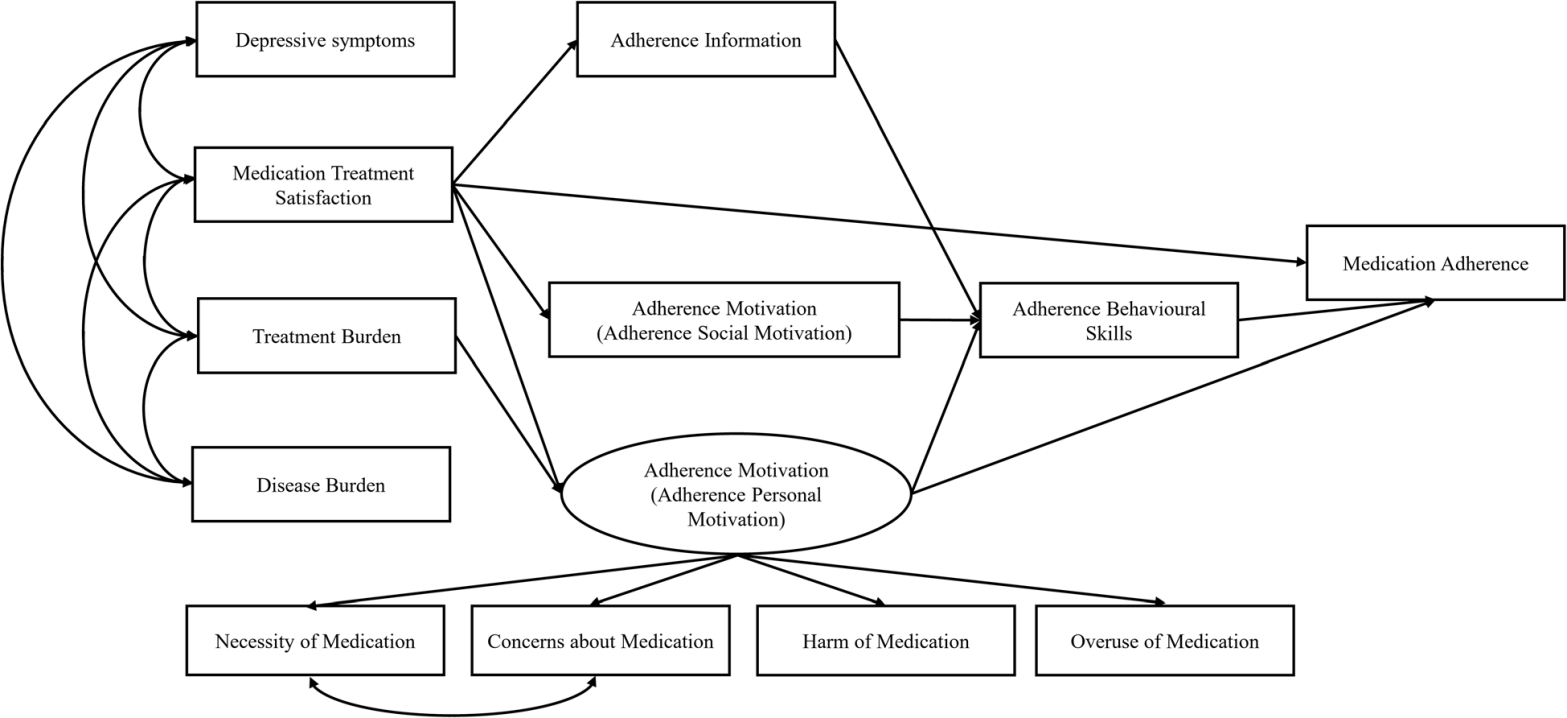
Theoretical framework of the study: the extended information-motivation-behavioural skills model of medication adherence

The intervention development, implementation, and evaluation are guided by a framework adapted from the Information-Motivation-Behavioural Skills (IMB) model of Fisher and Fisher [26]. The IMB model is a well-established social behavioural model for understanding and predicting health-related behaviours. In consideration of the complexity of patients living with multimorbidity, the IMB model was extended by including four interrelated variables (that is, disease burden, treatment burden, depressive symptoms, and medication treatment satisfaction) emerging from comprehensive literature reviews. The fitness of the extended IMB model (Fig. 2), as well as the relationships between model constructs, have been validated in our previous study [27, 28]. The extended IMB model demonstrated that in addition to adherence information, motivation, and behavioural skills, medication treatment satisfaction and treatment burden could also directly or indirectly impact medication adherence. Based on the model, it is proposed that individuals who are well informed, highly motivated, having skills to perform medication self-management, highly satisfied with medication treatment, and experiencing low treatment burden are more likely to enact and maintain adherence behaviour. The medication self-management intervention might be effective if intervention components focus on addressing each of the above behavioural determinants that affect medication adherence.

### Study setting

The trial will be conducted at three community health centres (CHCs) in Changsha, China. In urban China, CHCs serve as major primary care providers. Community general practitioners (GPs), nurses, public health physicians, pharmacists, and a few psychologists or social workers work collaboratively to provide essential public health services to community-dwelling populations including health education, medical treatment services, and chronic disease management in these CHCs [29]. All three recruited CHCs are teaching sites of the Xiangya Nursing School of Central South University and provide primary healthcare services covering a population of 40,000 to 60,000 people. The average number of patients visiting the centre per day ranges from 100 to 300 for each CHC, which provides an adequate number of participants for the trial.

### Participants

The participants of the trial are community-dwelling older patients with multimorbidity. Multimorbidity is defined using a cut-off of three or more chronic conditions as patients with at least three conditions are more likely to have complex needs and great utilisation of healthcare services [30]. In order to avoid ceiling effect and improve study power to detect changes in adherence, only participants with low medication adherence are considered eligible for the trial. Participants’ inclusion criteria are (1) 60 years old or over, (2) having at least 3 of the 38 chronic conditions (the list of chronic conditions is available at Additional file 1), (3) having at least one medication prescribed for a chronic condition over at least the 3 months prior to inclusion in the study, (4) nonadherence to medications, as defined by scoring 19 or lower on the 5-item Medication Adherence Report Scale (MARS-5) [31], (5) independently managing their medications (i.e., not rely on a carer), (6) able to speak and understand Chinese, (7) able and willing to receive phone calls, and (8) capable of providing a written informed consent to participate in the study.

Exclusion criteria are (1) having a life expectancy of less than 12 months, (2) being institutionalised in a nursing home or in a long-term care facility, (3) planning to move away from the community in the next 6 months, (4) cognitive impairment (Mini-cog scores < 4), and (5) currently participating in research involving chronic disease management. Patients with cognitive impairment are excluded because they may not be able to provide valid answers to the questionnaires.

### Recruitment

At least one community nurse will be recruited from each CHC to deliver the intervention. Nurses’ inclusion criteria are (1) community registered nurse with a junior college degree or above, (2) having at least 2 years of working experience in CHCs, (3) taking on chronic disease management in routine work, and (4) not planning to quit the job in the next 6 months. Nurses who are participating in other research studies on chronic disease management at the time of this study will be excluded. Nurses will recruit participants through approaching potentially eligible participants in waiting rooms in CHCs. Centre appointments by nurses will be arranged for interested participants and the principal investigator will determine the eligibility of participants, describe the study to them, obtain written consent forms, and collect baseline data. A one-week period will be allowed for participants to decide whether or not they wish to participate in the study. Participants’ reasons for non-participation will be recorded.

### Sample size estimation

Sample size calculation was performed using G*Power Version 3.1.9.4 [32]. Estimation is based upon the primary outcome, medication adherence, at 3 months of follow-up between the intervention and control group. Medication adherence is calculated as sum of the scores from each item using MARS-5. Based on our recent systematic review and meta-analysis on medication adherence interventions among older patients with multimorbidity (manuscript in preparation), a medium Cohen’s effect size (d) of 0.52 on medication adherence is estimated for a medication self-management intervention program. The required sample size will be 120 participants (60 in the intervention group and 60 in the control group) to attain an 80% power with a two-sided α of 0.05. Based on previous studies on older patients with multimorbidity, the attrition rate ranged from approximately 0% [20] to 12% [33]. Therefore, allowing for a 12% attrition rate, the final sample size required is 136 participants. If only 60% of participants agree to participate in the study, 227 participants need to be invited.

### Intervention group

The medication self-management intervention consists of three face-to-face education sessions and two weekly follow-up phone calls over 6 weeks. Intervention components are derived from an extensive review of the literature, including the related theoretical framework and current practice. Based on the extended IMB model of medication adherence, this intervention is designed to offer information related to medication treatments, motivate patients to adhere, help build medication self-management skills, and develop adherence improvement plans. The face-to-face sessions will include all of the following: exploration of medication treatment experience and expectation, medication knowledge education, motivation feedback tailored to the individual’s personal and social barriers to adherence, medication self-management skills building, and goal setting.

A patient-centred approach will be adopted considering the complex needs of patients with various combinations of chronic conditions. A comprehensive assessment of adherence problems will be firstly conducted to identify the factors that affect adherence, including how and why these factors contribute to poor adherence. Medication-related knowledge and skills will be provided based on individual treatments and barriers to adherence. Motivational interviewing techniques will be used for a better understanding of patients’ cognitive factors of adherence behaviour. Nurses will discuss with patients to explore their preferences and priorities in medication treatments, setting goals that are practical and acceptable for addressing their problems in medication use, and develop individualised adherence improvement plan to reach these goals. Taking patients’ complexity into account, strategies to reduce treatment burden will also be incorporated into intervention sessions, including prioritising medication and other self-management activities using shared decision making, providing communication skills with healthcare providers and encouraging patients to discuss with their physicians about burdensome regimens, exploring patients’ family and social network to support their self-care, and helping them to incorporate self-care activities into daily life.

Each face-to-face session will last approximately 30–40 min and will be delivered individually in the CHC by trained community nurses. The duration of each session can be adjusted by nurses in accordance with the patients’ barriers and problems to medication adherence. The three face-to-face sessions are designed to complete at 7–10-day intervals over 4 weeks. Before the first face-to-face session, nurses will review patients’ clinical health records. Patients will be asked to bring all medications to CHCs. After the conclusion of the last face-to-face session, education materials containing instructions and information on the name, purpose, side effects, and special tips for each usual chronic medication and medication skills will be provided to participants. After the face-to-face sessions, patients are followed up by two weekly phone calls. Each call lasted 10–20 min. The aim, content, and delivery strategy of each session are shown in Table 1.

**Table 1:** Overview of the Nurse-led Medication Self-management Intervention Sessions

Aim	Content	Delivery strategy
**1st face-to-face education session**		
• To identify patients’ problems and barriers to medication adherence. • To educate patients on the right information about medication-taking and adherence.	(1) Inform patients about the purpose and procedure of the whole intervention; Rapport building. (2) A structured patient-centred adherence assessment, covering a checklist with all the questions about patients’ medication experiences, including effectiveness and side effects of the drugs, treatment burden, and barriers to adhering to medications. (3) Educate patients on disease- and medication-related information and correct misinformation, including the name, dosage, frequency, timing, purpose of each medication, potential side effects, and special administration considerations. (4) Encourage patients to ask questions and confirm patients understand what they are told.	❖ Individualisation ❖ Teach-back technique
**2nd face-to-face education session**		
• To help patients change negative attitudes and beliefs about medication treatments and become motivated to it. • To help patients change negative perceptions of social norms for medication-taking and facilitate a positive attitude toward social support for adherence	(1) Empower patients with knowledge about the importance of medication adherence and the consequences of nonadherence. (2) Explore patients perceived social norms of adherence and the role of social support networks in supporting medication treatments and self-care. (3) Encourage patients to express their feelings and barriers to their family members and seek help where appropriate. (4) Provide a list of local resource information to patients (e.g., medical care institutions, psychological support organisations, and peer support teams).	❖ Motivational interviewing ❖ Mobilising social support
**3rd face-to-face education session**		
• To help patients develop skills and strategies to overcome practical barriers of their medication treatments. • To help patients set goals and develop a workable adherence improvement plan.	(1) Nurses will educate patients about six skills in medication self-management: 1. Identifying and coping with medication side effects; 2. Incorporating medication treatments into daily life; 3. Obtaining and updating medication adherence-related information; 4. Acquiring, self-cueing and self-administering medications; 5. Effectively communicating with healthcare providers; 6. Acquiring social and instrumental support for adherence. (2) Ask patients to select three skills they most want to learn or help most to take their medications; Discuss with patients about strategies on how to apply the three skills to their self-management. (3) Set goals and help make an individualised adherence improvement plan based on patients’ expectations and preferences.	❖ Planning coping responses ❖ Goal setting
**2 weekly follow-up phone calls**		
• To further explore the challenges and difficulties in medication self-management. • To provide feedback and suggestions according to patients’ performance.	(1) Ask whether patients can adhere to the medications in the last week and if not, the reasons for medication nonadherence will be asked and discussed with patients. (2) Ask whether patients can apply skills in medication self-management and encounter any challenges and concerns in medication-taking. (3) Further adherence education where needed. (4) Conclude what is discussed and encourage patients to contact healthcare providers when they have adherence problems.	❖ Active listening ❖ Personalised feedback

### Control group

Participants in the control group will continue to receive usual care from community GPs, nurses, public health physicians, pharmacists, and other healthcare providers in the CHC under the regular healthcare system of China. In China, community GPs are the primary providers and coordinators of care for patients with chronic conditions. GPs provide patients consultations and education regarding their diseases and treatments (typically clinician-centred) at each patient visit to the chronic disease clinic. Referral to hospital specialists will be made by GPs as needed. For patients diagnosed with hypertension and/or diabetes, community GPs are required to provide scheduled follow-up via home visits or telephone contact at least four times once a year. Community nurses and other healthcare providers assist GPs with chronic disease management.

In respect of medication management, community GPs will prescribe medication regimens, provide medication consultations (including instructions on the medications’ dosage, methods, and frequency), and adjust regimens where appropriate through discussion with patients. Community nurses’ involvement in medication management for patients with chronic conditions is comparatively little. No structural medication self-management education session will be provided for patients in usual care group.

### Nurses’ training

Intervention nurses will receive a training session from the principal investigator and a local nurse expert in motivational interviewing and geriatric care to standardise the intervention prior to the study. Firstly, nurses will receive a two-hour one-on-one training by the principal investigator. Nurses will be informed of the study objectives, procedure, detailed intervention protocol, and ethical considerations. Training sessions will involve medication self-management support, teach-back techniques, shared decision making, and basic motivational interviewing skills, e.g., posing open-ended questions, reflective listening, and expressing empathy [34]. Nurses will also receive a detailed study protocol and a manual containing instructions for delivering the intervention. Then, nurses will receive 70 min of video training on how to implement motivational interviewing techniques in the intervention. The videos are developed specifically for this program by the local nurse expert. Before the implementation of intervention, nurses’ acquisition of the above skills will be assessed by the principal investigator and the local nurse expert through a practicum with simulated patients to demonstrate their abilities to deliver the intervention.

### Outcome assessment

The researcher masked to the participant allocation will collect follow-up data through telephone interview. Participants will be assessed at T0, T1, and T2. Table 2 provides an overview of primary and secondary outcomes as well as covariates at each time point. Permission will be obtained from the original authors to use the scales for this study.

**Table 2:** Schedule of enrolment, interventions, and assessments

TIMEPOINT	STUDY PERIOD				
	Enrolment	Baseline	Intervention	Follow-up	
		T0		T1	T2
**ENROLMENT:**					
Eligibility screen	X				
Informed consent	X				
Allocation		X			
**INTERVENTIONS:**					
Intervention group			X		
Control group			X		
**ASSESSMENTS:**					
**Primary outcome**					
Medication adherence (MARS-5)		X		X	X
**Secondary outcomes**					
Medication knowledge (PKMUQ)		X		X	X
Medication beliefs (BMQ)		X		X	X
Medication social support (MSSS)		X		X	X
Medication self-efficacy (SEAMS)		X		X	X
Medication skills (self-developed scale)		X		X	X
Medication treatment satisfaction (TSQM)		X		X	X
Treatment burden (TBQ)		X		X	X
Quality of life (EQ-5D-5L)		X		X	X
Utilisation of healthcare services (six-item questionnaire)		X			X
**Potential covariates**					
Socio-demographics (Patients questionnaire)		X			
Number of prescribed medications (one-item question)		X			
Disease burden (CIRS-G)		X			
Depressive symptoms (PHQ-9)		X			

### Primary outcome

#### Medication adherence

The Chinese version of MARS-5 will be administered to assess medication adherence of the participants [31]. The MARS-5 is a self-reporting measure of unintentional and intentional medication non-adherent behaviours with a 5-point Likert scale response ranging from 1 = always to 5 = never. The total score of the MARS-5 ranges from 5 to 25, with a higher score representing higher adherence to medication. A total score less than 20 indicates nonadherence to medications [35]. The MARS-5 has been demonstrated a reliable and valid scale for measuring medication adherence across a range of chronic conditions (Cronbach’s alpha ranged from 0.68 to 0.89) [36–38]. The internal consistency reliability of the MARS-5 among patients with multimorbidity was 0.75 [39]. The association between MARS-5 and electronic monitoring-based adherence ranged from 0.42–0.67 among patients with multimorbidity [39]. The MARS-5 has also been validated in Chinese patients with various chronic conditions with good reliability (Cronbach’s alpha = 0.762), content validity, and criterion-related validity [35, 40, 41]. The Chinese version of the MARS-5 showed satisfactory internal reliability (Cronbach’s alpha = 0.908) among older patients with multimorbidity in our previous study [27, 28].

### Secondary outcomes

#### Medication knowledge

The Patients’ Perceived Knowledge in Medication Use Questionnaire (PKMUQ) is used to determine participants’ medication knowledge [42]. The PKMUQ comprises five items covering two dimensions: general knowledge in medication use and drug interaction knowledge. The response scale ranges from 1 = strongly disagree to 5 = strongly agree, and the response scores of all 5 items will be summed. Higher scores indicate a higher level of medication knowledge. The Chinese version of the scale has well content validity and showed to be reliable for the whole scale (Cronbach’s alpha = 0.647), the general knowledge subscale (Cronbach’s alpha = 0.912), and the drug interaction knowledge subscale (Cronbach’s alpha = 0.861) [43]. The test-retest reliability coefficients over a 2- to 4-week period for the whole scale and subscales were more than 0.7 [43]. The Chinese version of the PKMUQ showed good internal reliability (Cronbach’s alpha = 0.723) among older patients with multimorbidity in our previous study [27, 28].

#### Medication beliefs

Medication beliefs will be measured by using the Beliefs about Medication Questionnaire (BMQ). The BMQ is an 18-item self-reported questionnaire consisting of two subscales: the BMQ-Specific which evaluates representations of medications prescribed for personal use and the BMQ-General which evaluates beliefs about medications in general [44]. The BMQ-Specific subscale consists of two subscales: the 5-item Specific-Necessity subscale assesses beliefs about the necessity of medications and the 5-item Specific-Concerns measures concerns about the medications. The BMQ-General contains 8 items which include two 4-item subscales: the General-Harm subscale which evaluates beliefs about the harm of medication and the General-Overuse subscale which evaluates beliefs about overuse of medication by doctors [35]. Participants will be asked to rate the degree of agreement with each statement. A 5-point Likert scale ranging from 1 = strongly disagree to 5 = strongly agree is used. A higher score indicates stronger beliefs about the corresponding concepts in each subscale. The Chinese version of BMQ with proven good reliability and validity has been widely used. The Cronbach’s alpha of overall Chinese-version questionnaire was 0.738. The factor analysis explained 55.42% of the total variance, indicating an acceptable construct validity [45]. The Chinese version of the BMQ showed acceptable internal reliability (Cronbach’s alpha = 0.639) among older patients with multimorbidity in our previous study [27, 28].

#### Medication social support

Medication social support will be assessed by the self-administered 8-item Medication-Specific Social Support Questionnaire (MSSS). Participants will be asked how often others help them with their medications over 3 months [46]. Each item is scored on a 5-point scale ranging from 0 = never to 4 = very often. A single score is derived from the mean of all items, with a higher score indicates more medication social support. A previous study has demonstrated the scale’s reliability and validity [46]. The Chinese version of the MSSS showed satisfactory internal reliability (Cronbach’s alpha = 0.878) among older patients with multimorbidity in our previous study [27, 28].

#### Medication self-efficacy

The Self-Efficacy for Appropriate Medication Use Scale (SEAMS) will be used to determine participants’ medication self-efficacy [47]. The SEAMS consists of 13 items that evaluate participants’ confidence in taking medications under various challenging circumstances. Each item has a 3-point scale ranging from 1 = not confident to 3 = very confident. The score of scale ranges from 13 to 39, with a higher medication self-efficacy by a higher score. The Chinese version of SEAMS is a reliable and well-validated measure to assess medication self-efficacy (Cronbach’s alpha = 0.915) [48]. Two factors were extracted by principal component analysis, which accounted for 60.86% of the total variance. A significant positive correlation was found between the Chinese version of SEAMS and general self-efficacy scale (*r* = 0.531, *p* < 0.001). The 10-day test-retest reliability of the scale was also acceptable (*r* = 0.642) [48]. The Chinese version of the SEAMS showed satisfactory internal reliability (Cronbach’s alpha = 0.935) among older patients with multimorbidity in our previous study [27, 28].

#### Medication skills

A medication skills assessment scale is developed for the current study and used to determine how hard or easy for patients to apply skills in medication self-management. The scale contains six items related to the skills which participants will receive in the intervention sessions. Participants respond to questions using a 5-item scale ranging from 1 = very hard to 5 = very easy. The score from each item can be summed to yield a total score and a higher score indicates a higher level of medication skills. The reliability and feasibility of the assessment scale will be tested prior to the initiation of the intervention, which will be reported elsewhere.

#### Medication treatment satisfaction

Medication treatment satisfaction will be assessed using the Treatment Satisfaction Questionnaire with Medication (TSQM) Version II [49]. The 11-item TSQM Version II scale comprises four dimensions: effectiveness, convenience, side effects, and global satisfaction. Response options are in 5- or 7- point Liker-type format. Item scores can be summed into a total score ranging from 0 to 100, with a higher score indicating higher convenience, better effectiveness, higher global satisfaction, and fewer side effects. The Cronbach’s alpha for the Chinese version of TSQM Version II was 0.707 and ranged from 0.847 to 0.961 for each subscale [43]. The test-retest reliability coefficients over a 2- to 4-week period were 0.982 for the whole scale and ranged from 0.930 to 0.995 for each subscale. Three factors were extracted by factor analysis to contribute to 73.26% of total variance [43]. The Chinese version of TSQM Version II showed good internal reliability for the total scale (Cronbach’s alpha = 0.860) and each subscale (Cronbach’s alpha ranged from 0.735–0.983) among older patients with multimorbidity in our previous study [27, 28].

#### Treatment burden

The treatment burden will be measured by the 15-item Treatment Burden Questionnaire (TBQ) [50, 51]. It assesses a broad range of behavioural and emotional burden in the procedure of undertaking and engaging treatments associated with medication-taking, self-care activities, and healthcare costs. The scale consists of three domains of treatment burden: (1) medication regimen (4-item), (2) navigating the healthcare system (5-item), (3) lifestyle changes, social and financial impacts (6-item) [52]. Each item is scored on an 11-point Likert scale, ranging from 0 = not a problem to 10 = big problem. Scores are summed to derive a total score ranging from 0 to 150 with a higher score indicating a higher level of treatment burden. The TBQ is a valid and reliable measure for patients with chronic conditions across several countries with the Cronbach’s alpha = 0.89 [50, 51]. The Chinese version of TBQ has been validated among patients with multimorbidity [53]. The Cronbach’s alpha was 0.842 and 2-week test-retest reliability was 0.830. Spearman’s correlations were over 0.4 for all items indicating adequate internal construct validity. Results of factor analysis showed a three-factor structure model with adequate goodness-of-fit. The Chinese version of TBQ showed satisfactory internal reliability (Cronbach’s alpha = 0.886) among older patients with multimorbidity in our previous study [27, 28].

#### Quality of life

Participants’ quality of life will be measured by EuroQol-5D-5L (EQ-5D-5L). It has been widely used in measuring generic health status, population health surveys, and health economic evaluations. The EQ-5D-5L consists of a five-dimension descriptive system (i.e., mobility, self-care, usual activities, pain/discomfort, and anxiety/depression) and a visual analogue scale (VAS) [54]. The five-dimension descriptive system is described at five levels (no problems, slight problems, moderate problems, severe problems, and extreme problems). The VAS is participants’ self-report health status ranging from 0 (worst imaginable health state) to 100 (best imaginable health state). A summary index score will be calculated based on the value set for the Chinese population developed by Luo et al. [55]. The EQ-5D-5L has been validated in the Chinese population (Cronbach’s alpha = 0.624). Factor analysis for construct validity showed two factors explaining 61.21% of total variance [56].

#### Utilisation of healthcare services

The utilisation of healthcare services will be assessed via a self-reported questionnaire. Information about the number of CHC visits, number of outpatient consultations, number of emergency department visits, number of hospital admissions, days in the hospital, and medical costs over the last 3 months will be collected at baseline and at 3 months after the intervention.

### Potential covariates

#### Socio-demographic characteristics and the number of prescribed medications

Socio-demographic characteristics will be collected at baseline from patients. Patients will be asked about age, gender, educational level, marital status, insurance status, monthly income, and the number of prescribed medications.

#### Disease burden

The Cumulative Illness Rating Scale-Geriatric (CIRS-G) will be used to estimate disease burden [57]. It rates the severity of diseases across 14 organ systems on a 5-Likert point that ranges from 0 = no problem to 4 = extremely severe. The total score is the sum of each of the individual system score, with a higher score representing higher disease burden. The researcher will score the severity of diseases in each system according to the guideline for scoring the CIRS-G developed by Salvi et al. [58]. The scale and the scoring guideline have both been translated into Chinese and validated among Chinese older people [59]. CIRS-G has been recommended to be used for evaluating multimorbidity in older patients by Chinese experts [60].

#### Depressive symptoms

Depressive symptoms will be measured by the Patient Health Questionnaire-9 (PHQ-9), which is the most commonly used instrument for screening depression [61]. Participants will indicate how often each statement applied to them in the last 2 weeks on a 4-point Likert scale ranging from 0 = not at all to 3 = nearly every day. Items are summed to obtain a total score ranging from 0 to 27. A score of 10 or more is used to indicate depression conditions [62]. The PHQ-9 is a validated instrument with good internal consistency among the general Chinese population (Cronbach’s alpha = 0.86). The 2-week test-retest coefficient of the Chinese-version PHQ-9 was 0.86. Construct validity was supported by one factor that accounted for 48.9 of total variance. The PHQ-9 also had good concurrent validity with the self-rating depression scale (*r* = 0.29, *p* < 0.001) and with subscales of the 36-item short form health survey (r ranged from − 0.11 to − 0.47, *p* < 0.001) [63]. The Chinese version of PHQ-9 showed good internal reliability (Cronbach’s alpha = 0.759) among older patients with multimorbidity in our previous study [27, 28].

### Randomisation, allocation concealment and blinding

Participants per CHC will be randomly allocated to either the intervention or control group. Block randomisation stratified by site with a block size of six will be used to ensure an equal number of participants in the intervention and control group in each CHC. The randomisation and group assignment will be conducted by an independent research assistant (a nurse with a master’s degree) who is not involved in study design and intervention delivery. The randomisation sequence will be generated by using the website Randomization.com (http://www.randomization.com). After completion of baseline data collection, the research assistant blinded to the allocation order will assign the treatment group using sealed opaque envelopes based on the order of recruitment and inform participants of the study group to which they have been assigned. The intervention nurse will receive names and contact details of the participants allocated in the intervention group. This study is single-blinded. Nurses and participants are not feasible to be blinded to the group allocation because of the nature of the intervention. The researcher collecting baseline and follow-up data will be kept blind to participant allocation.

### Intervention fidelity and contamination

The principal investigator will be responsible for monitoring the procedure of the whole study. The intervention logbook will be used by participating nurses to record the number, duration, and administration date of face-to-face sessions and follow-up phone call sessions. To ensure that the delivery of motivational interviewing is consistent with its principals, all motivational interviewing sessions will be audiotaped if permitted by the participant. A random 20% sample will be reviewed using Motivational Interviewing Treatment Integrity Coding Manual 4.2.1 [64] by the local motivational interviewing nurse expert throughout the duration of the intervention. The principal investigator will meet with intervention nurses to discuss the experiences and difficulties to deliver the intervention sessions and provide feedback weekly. Intervention logbooks will also be examined weekly by the principal investigator and further training for nurses will be considered if the delivery of the intervention is not per protocol.

Contamination may occur whereby participants in the control group also receive usual care from trained nurses or interact with participants in the intervention group. However, it is expected that the current study has a low risk of contamination because (1) patients with chronic diseases predominately receive medication management by GPs in China, the involvement of community nurses in medication treatment and consultation is rare, (2) the multifaceted interventions with individualised cognitive behaviour component cannot easily be transferred from one participant to another. Also, the following strategies will be introduced to minimise contamination. First, participants and nurses will be provided with clear information on the nature and purpose of the study. Second, researchers will ask participants not to share information with other participants and keep the education materials to themselves until the end of the study. Finally, only intervention nurses will receive training to deliver the intervention and they will be requested not to discuss the intervention with their colleagues who might be involved in the control group. At the end of the trial, participants in the control arm will be asked whether they have received any information or education on their medications, causal average effect analysis will be considered if high degree contamination occurs [65].

### Statistical analysis

All data will be analysed using IBM SPSS Statistics 25.0 software. Descriptive statistics will be used to describe and compare socio-demographic data on participants between the intervention and control group. Continuous variables that follow a normal distribution will be expressed as means and standard deviations, whereas categorical variables will be presented as frequency counts and percentages. Median values and interquartile ranges will be calculated for continuous data with a skewed distribution. Mann-Whitney U-test or t-test will be performed for continuous data to compare baseline characteristics between groups, and Chi-squared test or Fisher’s exact test will be used for categorical variables. The distributions of the outcome variables will be evaluated, and normalising transformation will be applied as needed.

Primary and secondary outcomes will be analysed following the intention to treat principle at the individual participant level. Changes in repeated outcome measures across different time points between the intervention and control group will be analysed using generalised estimating equation models with adjustment for potential confounders of the baseline variables. All statistical tests are two-sided, and *p* < 0.05 will be statistically significant.

### Data management

The research data including audio recordings and participants’ personal information will be recorded in two different files. All research-related data will be linked with a study ID number with no identifying information. Research data will be stored on a password-protected computer and backed up on a password-protected hard drive. Original questionnaires and tapes will be locked in a secure cabinet, with limited access by designated research team members only.

### Trial status

At the time of submission of this manuscript (April 2020), participant recruitment has not yet commenced and will start in June 2020. It is expected that recruitment will complete by August 2020.

## Discussion

Although the high prevalence and adverse consequences of medication nonadherence among older patients with multimorbidity have been well established in current research, intervention programs designed to improve medication adherence are limited in these complex patients. Self-management support has increasingly been recognised as an effective strategy for patients with chronic diseases in primary care [66]. An evidence-based and theory-informed medication self-management intervention designed specifically for older patients suffering from multimorbidity has been developed, which would enable patients to manage their medications more effectively by improving their self-management capacity in terms of medication knowledge, motivation, and skills. This protocol describes the design of a RCT to determine whether the intervention delivered by nurses leads to improvement in medication adherence and health outcomes in community settings.

The novelty of the medication self-management intervention lies in the combination of patient education, cognitive-behaviour change, self-management skills training, and goal setting. Various strategies including motivational interviewing and individualised feedback are adopted to tailor patient-centred care, given the multifaceted and interactive effects of personal and condition factors that affect adherence behaviour for older patients with multimorbidity [67]. A systematic review suggested that self-management support interventions should be tailored to patient needs and include combination of various strategies such as patient education, behavioural change, and goal setting [68]. Guidelines on multimorbidity management have also highlighted the importance of addressing a person’s individual needs, preferences and goals in the care of adults with multimorbidity [69, 70]. In present study, a comprehensive adherence assessment can not only identify personal facilitators and barriers of medication adherence but help nurses to consider individual patient needs and expectations. Motivational interviewing, a collaborative conversation style, is used to further understand patient situation and elicit and strength motivation for adherence behaviour change. To enable patients to be active partners in their self-management, patients will be involved in goal settings and the development of the adherence improvement plan. Patients’ treatment complexity will be considered to develop a realistic and feasible care plan. Individualised feedback will also be provided to patients after face-to-face education sessions through two follow-up phone calls, which are helpful to hone patients’ self-management and problem-solving skills.

Assessing, reducing and managing treatment burden cannot be ignored in respect of care for multimorbidity. Patients with multimorbidity are required to invest much time and efforts to implement medical treatment prescriptions, which creates a considerable burden and thus reduces their capacity to enact self-care. In our previous study [27, 28], treatment burden was demonstrated to have a negative impact on medication adherence through affecting adherence personal motivation and behavioural skills. Therefore, several strategies to assess and reduce the treatment burden are incorporated into the intervention. However, it is hard to say whether the intervention will result in a significant reduction of treatment burden because various patient-, treatment-, and organisational-related factors can contribute to treatment burden, such as burdensome self-care, physical limitations, financial constraints, patient-provider relationships, and organisational factors associated with the healthcare system [71]. Also, patients who are overwhelmed by treatment regimens might feel difficult and burdensome to adhere to recommendations and skills provided in intervention sessions, which can reduce the potential efficacy of the intervention. The impact of patients’ treatment burden on the intervention implementation and effects will be considered and analysed in this study. Moreover, the study specifically focuses on participants who have low adherence to their medications. A high level of adherence at baseline may limit the potential to detect changes in adherence outcomes. Participants with adherence problems therefore are most likely to gain benefits from the current intervention.

Another strength of the study is that the study design, implementation, and evaluation is guided by a validated, well-established model, which was adapted specifically for older patients with multimorbidity. Each construct in the model will be measured using patient-reported outcomes to identify the contribution of each component from the multifaced intervention and to present how the intervention might work, which provides a great opportunity to generalise findings to other populations and settings.

Finally, the nurse’s role in medication management for older patients with multimorbidity will be explored. A Cochrane review found that trained nurses in primary care might provide better quality care, achieve better health outcomes, as well as higher levels of patient satisfaction, compared to doctors [72]. Nurses are well-positioned to provide health promotion, care coordination and deliver self-management support [73, 74]. However, medication management interventions are usually delivered by doctors or pharmacists, nurses currently represent an underestimated and underutilised force in this field [17, 74]. The proposed study will provide information about strategies and procedures for nurses to conduct medication self-management support in primary care practice.

There are some potential limitations that should be considered in the current study. First, the primary outcome, medication adherence, will be evaluated by a self-reported measure. Although it is the most practical way due to its low cost and easy to implement in primary care practice, it tends to overestimate adherence because of recall bias and social desirability bias. Other widely used methods for measuring adherence include electronic adherence monitoring and pharmacy claims data. Electronic adherence measurements, which are recognised as the gold standard of medication adherence measurements by some researchers [39], are expensive and susceptible to overestimate adherence as it assumes that patients take medications as prescribed when opening electronic pill bottles. Pharmacy claims data from community electronic health records are not practical and accurate as well in China. This method requires a centralised electronic system containing all pharmacy refill data of the patient, which is unavailable in China. It is common for patients to obtain their medications not only from CHCs, but also from many other ways, such as hospitals, drug stores, or online pharmacies. Moreover, patients with multimorbidity are often prescribed same medications from multiple clinicians. Therefore, measurement of medication adherence using pharmacy claims data of CHCs may not reflect the medications patients actually ingest and are therefore not appropriate in our study. Second, older patients with multimorbidity suffering from great disease and treatment burden are at high risk of medication nonadherence and thus, in most need of self-management support. However, participants who are already busy with self-care, work, or family obligations might not afford the time and energy to attend the trial, which leads to potential selection bias. Reasons for refusal to participate in the study will be recorded to provide information on study participants.

A small pilot study has been conducted before the start of the trial from Dec 2019 to Jan 2020. Twenty-eight older patients with multimorbidity recruiting from one CHC in China were enrolled. The full analysis of the pilot trial is ongoing. Preliminary results showed that the intervention program was feasible and acceptable and had the potential to have positive effects on medication adherence. Findings from the pilot study may inform changes in the study implementation and evaluation. Any change made to the protocol will be reported in later published articles about this trial.

In conclusion, this study will provide insights into strategies to support medication self-management and adherence for older patients with multimorbidity. If effective, the nurse-led medication self-management intervention could be integrated into the current primary care practice to enhance medication adherence and maximise the efficacy of medication treatments.

## Supplementary Information

**Additional file 1.** List of the 38 chronic conditions in multimorbidity eligibility screen.

## Data Availability

Not applicable.

## Abbreviations

BMQ: Beliefs about Medication Questionnaire
CHC: Community Health Centre
CIRS-G: Cumulative Illness Rating Scale-Geriatric
EQ-5D-5L: EuroQol-5D-5L
GP: General practitioner
IMB: Information-Motivation-Behavioural Skills
MARS-5: 5-item Medication Adherence Report Scale
MSSS: Medication-Specific Social Support Questionnaire
PHQ-9: Patient Health Questionnaire-9
PKMUQ: Patients’ Perceived Knowledge in Medication Use Questionnaire
SEAMS: Self-Efficacy for Appropriate Medication Use Scale
T0: Baseline
T1: Immediately post-intervention
T2: 3-month post-intervention
TBQ: Treatment Burden Questionnaire
TSQM: Treatment Satisfaction Questionnaire with Medication
VAS: Visual Analogue Scale
