# Supplementary material for: A community-based nurse-led medication self-management intervention in the improvement of medication adherence in older patients with multimorbidity: protocol for a randomised controlled trial
Source: BMC Geriatr. 2021 Mar 2;21:152. doi: 10.1186/s12877-021-02097-x (PMC7923480; doi:10.1186/s12877-021-02097-x)
Supplement: Supplementary file 1 — Additional file 1. List of the 38 chronic conditions in multimorbidity eligibility screen. [file 12877_2021_2097_MOESM1_ESM.docx]

**Additional file 1.** List of the 38 chronic conditions in multimorbidity eligibility screen adapted from Wang et al. [1]

| Chronic conditions (Simplified Chinese) |
| --- |
| Hypertension (高血压) |
| Chronic painful condition (慢性疼痛) |
| Inflammatory connective tissue disorders (炎性结缔组织疾病) |
| Diabetes (糖尿病) |
| Lipid disorder (血脂异常) |
| Dyspepsia and gastroenteritis (消化不良和肠胃炎) |
| Coronary heart disease (冠心病) |
| Chronic obstructive pulmonary disease (慢性阻塞性肺疾病) |
| Stroke and cerebrovascular disease (中风/脑血管疾病) |
| Nephritis and chronic kidney disorder (肾炎/慢性肾病) |
| Gallbladder/Spleen diseases (胆囊/脾脏疾病) |
| Peripheral vascular disease (周边血管疾病) |
| Cancer (癌症) |
| Multiple sclerosis/neurological disorder (多发性硬化/神经系统疾病) |
| Bronchiectasis (支气管扩张) |
| Glaucoma / Cataract (青光眼/白内障) |
| Asthma (哮喘) |
| Chronic sinusitis (慢性鼻窦炎) |
| Viral Hepatitis (病毒性肝炎) |
| Chronic pharyngitis/Laryngitis (慢性咽炎/喉炎) |
| Diverticular disease of intestine (肠道憩室病) |
| Thyroid disorders (甲状腺疾病) |
| Inflammatory bowel disease (炎症性肠病) |
| Hearing loss/Tinnitus (听力受损/耳鸣) |
| Blindness/Low vision (失明/视力低下) |
| Psoriasis/eczema (牛皮癣/湿疹) |
| Anaemia (贫血) |
| Prostate disorders (前列腺疾病) |
| Migraine (偏头痛) |
| Chronic liver disease (慢性肝病) |
| Depression (抑郁) |
| Epilepsy (癫痫) |
| Anxiety & other stress related disorders (焦虑/其他压力相关疾病) |
| Parkinson’s disease (帕金森病) |
| Irritable bowel syndrome (肠易激综合征) |
| Constipation (便秘) |
| Chronic tonsillitis (慢性扁桃体炎) |
| Anorexia/bulimia (厌食症/贪食症) |

**Reference**:

1. Wang HHX, Wang JJ, Wong SYS, Wong MCS, Li FJ, Wang PX, et al. Epidemiology of multimorbidity in China and implications for the healthcare system: Cross-sectional survey among 162,464 community household residents in southern China. BMC Med. 2014;12:188.
